# Management of an Unusual Post-arthroscopic Complication: Subquadricipital Hematoma in the Presence of Complete Suprapatellar Plica

**DOI:** 10.7759/cureus.22450

**Published:** 2022-02-21

**Authors:** Can Yener, Mesut Demirkoparan, Elcil Kaya Bicer, Semih Aydogdu

**Affiliations:** 1 Department of Orthopaedics and Traumatology, Ege University Faculty of Medicine, Izmir, TUR

**Keywords:** plica, suprapatellar, complete, hematoma, subquadricipital, post-arthroscopic

## Abstract

Complete suprapatellar plica is a rare congenital anomaly that separates the suprapatellar bursa from the knee joint cavity. Although the pathological incidence of this condition is not known, it can cause patellofemoral symptoms, anterior knee pain, and rarely hemarthrosis. We report a patient with a hematoma in an unusual location just three weeks after an arthroscopic procedure, associated with a complete suprapatellar plica undetected during primary surgery. The hematoma was not in the synovial cavity, rather between the quadriceps tendon and the synovial membrane and presenting with pain and catching. This unusual location has not been reported before. Repeat arthroscopic surgery with drainage of hematoma and plica resection relieved the symptoms.

## Introduction

Knee plicae are embryological synovial membrane remnants [1]. The incidence of the suprapatellar type has been reported to be approximately 90% [2]. Suprapatellar plicae can be seen in different forms. Although the most common is the incomplete arch type, complications are most commonly associated with complete suprapatellar plicae [3,4]. These suprapatellar plicae completely divide the joint into two separate compartments. The pathophysiology of the complete suprapatellar plica is thought to be that the plica loses its elasticity and becomes hypertrophic as a result of inflammatory or traumatic processes [5]. Besides, the hydraulic theory with a one-way valvar mechanism that causes fluid accumulation in the upper compartment is thought to play a role in the emergence of symptoms [6].

In the previously reported cases of symptomatic plica, a traumatic or spontaneous process was mentioned [7,8]. Symptomatic suprapatellar plica presents with anterior knee pain and patellofemoral symptoms [9]. Fluid accumulation in the compartment above the suprapatellar plica and associated symptoms have been reported in previous case reports and series [7,8]. However, to the best of our knowledge, post-arthroscopy formation of a hematoma located between the quadriceps tendon and the synovial membrane in the presence of a complete suprapatellar plica has not been reported before.

## Case presentation

**Figure 1:**
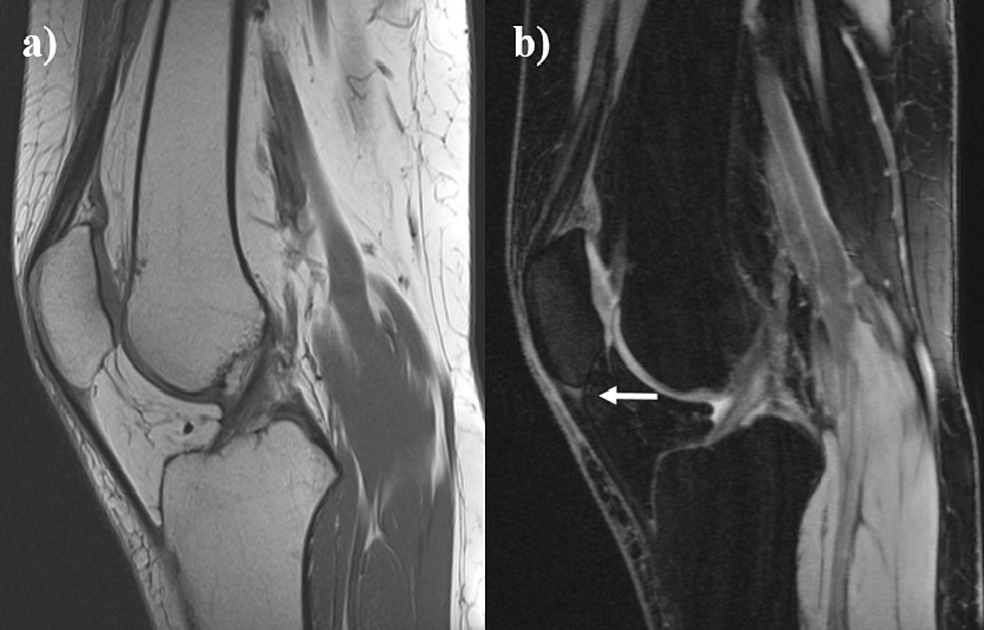
MRI images of the patient diagnosed with patellar tendinitis a) T1-weighted image b) T2-weighted image shows increased signal intensity at the inferior pole of patella (arrow).

A 34-year-old woman was presented with chronic infrapatellar knee pain after a fall on her left knee five months ago. Routine radiographs were unremarkable. The patient had persistent pain at the inferior border of the patella, increasing with palpation and activities. Patellar tendonitis was diagnosed upon patient’s symptoms, physical examination, and MRI findings (Figure 1).

**Figure 2:**
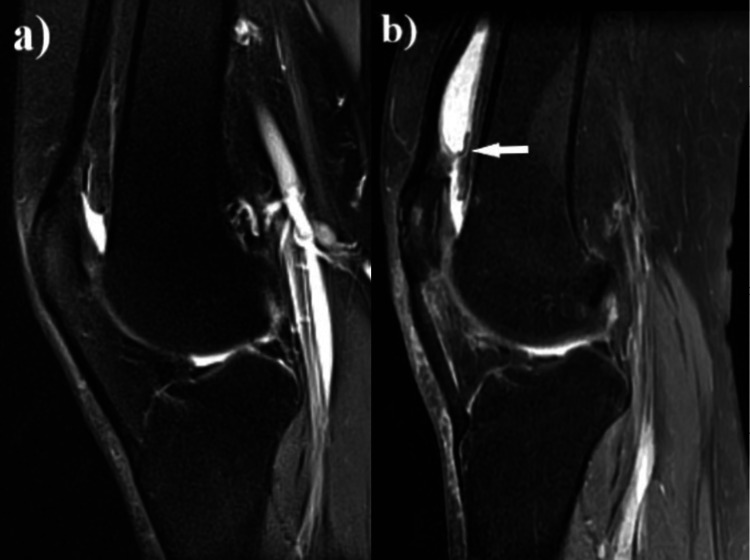
MRI images of the patient a) MRI performed for patellar tendonitis before the first operation. No pathology detected in the suprapatellar region, except no-passage of synovial fluid to the suprapatellar area; b) MRI image of the mass in the suprapatellar pouch before the re-arthroscopy. Liquid intensity mass isolated from the joint cavity by a band-like structure (arrow).

Conservative treatment modalities were performed for four months with rest, activity modification, exercises, and later on with platelet-rich plasma injection and dry needling. Since the patient could not get satisfactory pain relief, a minimally invasive surgical intervention was required. Diagnostic arthroscopy showed no meniscal, ligamentous, or chondral pathology. Since no pathology was expected in the suprapatellar region, that area was not evaluated in detail (Figure 2A). Removal of the injured portion of the tendon and patellar drilling were performed. The patient was symptom-free after surgery.

Three weeks after surgery, she experienced sudden-onset suprapatellar knee pain that started while she was getting up from sitting. Within a few days, a mobile, firm, palpable fullness was formed in the suprapatellar pouch, extending laterally. The mass was prominent in flexion and was causing pseudolocking combined with snapping. The patellar tap test was negative. The patient had no history of disease that would predispose to bleeding tendency. There were no signs of knee infection such as warmth or redness of the skin over the knee and complete blood cell count, C-reactive protein, and erythrocyte sedimentation rates were within normal limits. On MRI, a well-circumscribed, fluid-intensity collection was observed in the suprapatellar pouch, separated from the joint cavity by a band-like structure (Figure 2).

**Figure 3:**
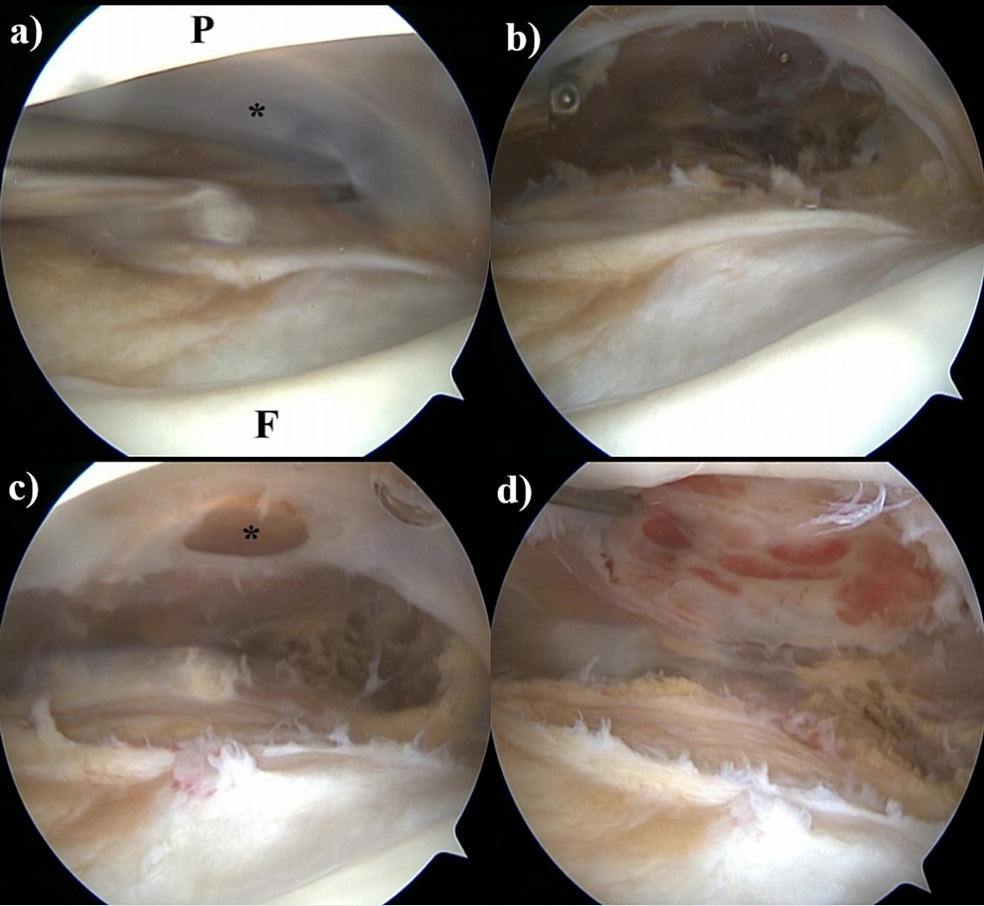
Arthroscopy images of the patient a) Arthroscopy view of the complete suprapatellar plica (P: patella, F: femur, asterisk: complete plica); b) complete plica is resected; c) the synovial membrane that encircles the overlying hematoma is perforated; d) the membrane surrounding the hematoma is completely resected and the hemorrhagic appearance of new cavity floor is seen.

This structure was not noticed in the previous MRI and during arthroscopy performed for patellar tendonitis. Despite the conservative management with general anti-inflammatory medication, compressive bandaging, and cold therapy, the collection did not regress, and arthroscopy was planned to alleviate the symptoms. Pre-arthroscopy aspiration of the collection revealed a hemorrhagic nature. Complete aspiration was not performed in order to be able to determine the location of the collection during the arthroscopic intervention. This arthroscopic examination revealed that meniscal structures, cartilage, and ligaments were intact. The complete suprapatellar plica was viewed and perforated with a shaver blade (Figure 3).

At this point, the hemorrhagic fluid was expected to drain, but it was not (Video 1). The plica was completely resected. The collection was still palpable, and its location was determined on arthroscopy with the aid of palpation. Sudden hematoma drainage was observed only after the perforation of the synovial membrane under the quadriceps tendon (Video 1).

**Video 1:** Arthroscopy video of the patient Arthroscopic drainage of an isolated suprapatellar hematoma. While it was expected to drain after complete suprapatellar plica resection, it was not. The hematoma drained suddenly by perforating the synovial membrane below the quadriceps tendon. The membrane surrounding the hematoma was resected.

This membrane that encircled the hematoma was completely resected. The fullness disappeared. No tumoral tissue was detected in the pathology samples. No complaints and no recurrence were noted postoperatively.

## Discussion

The complete suprapatellar plica is usually thought to be asymptomatic [9]. However, it can present with anterior knee pain and patellofemoral symptoms [10]. These symptoms usually begin with minimal trauma or sudden knee movements and increase with flexion [7]. In cases with persistent pain and mechanical symptoms, surgery is frequently required [11]. Surgical plica resection is recommended in pathological suprapatellar plica syndrome that does not respond to conservative treatment [11].

Complete suprapatellar plicae can also present as a mass lesion, less frequently mentioned in several case reports [7,8,12-18]. These cases usually appeared after minimal trauma or spontaneously. In our case, the symptoms also started after a minimal trauma such as sudden getting up from sitting. However, the occurrence of this complication after arthroscopic surgery can also be explained by the increase in intra-articular pressure due to the residual fluid, although the irrigation fluid was aspirated after arthroscopy as routinely. This may have caused bleeding in the subquadricipital area by increasing the pressure in the suprapatellar pouch aggravated by sudden getting up from sitting. Also, the increase in pressure may have changed the elasticity of the plica and caused bleeding at its adhesion to the membrane under the quadriceps. The complete suprapatellar plica complicated with hematoma formation just after an arthroscopy procedure has not been reported previously.

The incidence of complete suprapatellar plica has been reported to be 4-20% during routine arthroscopic surgery [3,19]. While the incidence of pathological complete suprapatellar plica is unclear, isolated synovial fluid accumulation, bursitis, chondrocalcinosis, and osteochondromatosis in the suprapatellar pouch due to the complete plica have been reported in previous publications [7,8,12-18]. In this case, as well, MRI images disclosed an isolated collection in the suprapatellar pouch, separated from the joint cavity by the complete suprapatellar plica. Aspiration prior to re-arthroscopy confirmed hemorrhagic content. During the re-arthroscopy, after perforation and resection of complete suprapatellar plica, we could not obtain any hemorrhagic material but only after having shaved and perforated the anterior wall of suprapatellar cavity hemorrhagic content drained in a flash inside the joint cavity. In this case, the complete suprapatellar plica was complicated by a hematoma located in the extra-articular location, not in the suprapatellar pouch as in previous reports [7,8,15,16]. Unlike these reports, only in our case, the symptoms occurred after arthroscopy. Therefore, the unexpected location of the collection may be arthroscopy-related. Considering that the symptoms started three weeks after the first arthroscopy and after sudden getting up from sitting, the aforementioned increased intra-articular pressure or fibrotic changes in the plica were thought to be more probable causes than direct injury with arthroscopic instruments.

## Conclusions

We report this unique post-arthroscopic complication presenting with a hematoma at an unexpected location in the presence of a complete suprapatellar plica and fullness. Surgery by re-arthroscopy relieved the symptoms. Therefore, it should be kept in mind that under similar circumstances, a hematoma in an unexpected location may be overlooked. In order to prevent this complication, more care may be taken to aspirate the residual fluid after arthroscopy.
